# Research on Hydrogen Production from Ammonia Decomposition by Pulsed Plasma Catalysis

**DOI:** 10.3390/molecules30051054

**Published:** 2025-02-25

**Authors:** Yuze He, Neng Zhu, Yunkai Cai

**Affiliations:** 1 School of Automobile and Traffic Engineering, Wuhan University of Science and Technology, Wuhan 430081, China; 18907147646@163.com; 2 School of Automotive Engineering, Hubei University of Automotive Technology, Shiyan 442002, China; 3 Key Laboratory of Automotive Power Train and Electronics (Hubei University of Automotive Technology), Shiyan 442002, China

**Keywords:** plasma, hydrogen production from ammonia decomposition, synergistic effect, nanosecond pulse discharge

## Abstract

Driven by dual-carbon targets, marine engines are accelerating their transition towards low-carbon and zero-carbon. Ammonium–hydrogen fusion fuel is considered to be one of the most promising fuels for ship decarbonization. Using non-thermal plasma (NTP) catalytic ammonia on-line hydrogen production technology to achieve hydrogen supply is one of the most important means to guarantee the safety and effectiveness of hydrogen energy in the storage and transportation process. However, the efficiency of ammonia catalytic hydrogen production can be influenced to some extent by the presence of several factors, and the reaction mechanism is complex under the conditions of ship engine temperature emissions. This makes it difficult to realize the precise control of plasma catalytic hydrogen production from ammonia technology under temperature emission conditions, thus restricting an improvement in the ammonia conversion rate. In this study, a kinetic model of hydrogen production from ammonia catalyzed by NTP was established. The influencing factors (reaction temperature, pressure, N_2_/NH_3_ ratio in the feed gas) and mechanism path of hydrogen production from ammonia decomposition were explored. The results show that the increase in reaction temperature will lead to an increase in the ammonia conversion rate, while the ammonia conversion rate will decrease with the increase in reaction pressure and N_2_/NH_3_ ratio. When the reaction temperature is 300 K, the pressure is 1 bar, the feed gas is 98%N_2_/2%NH_3_, and the ammonia conversion rate is 16.7%. The reason why the addition of N_2_ is conducive to the hydrogen production from NH_3_ decomposition is that the reaction N_2_(A3) + NH_3_ => N_2_ + NH_2_ + H, triggered by the electron excited-state N_2_(A3), is the main reaction for NH_3_ decomposition.

## 1. Introduction

**Figure 1:**
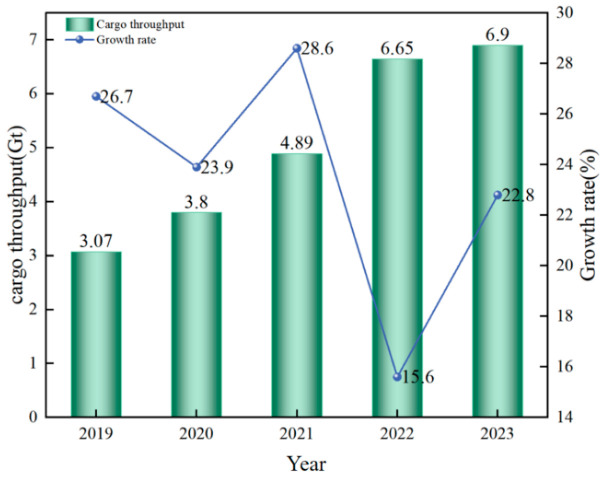
Cargo throughput in Hubei and its growth rate from 2019 to 2023.

In recent years, the issue of global climate change has become increasingly severe, and greenhouse gas emissions have become a focal point of international concern. According to a report by the International Maritime Organization (IMO), the shipping industry is one of the significant sources of global carbon emissions, accounting for 6% to 11% of the total emissions in the transportation sector [1]. As the world’s largest shipbuilding and ship-using country, China bears important responsibilities in promoting the green and low-carbon transformation of ships. Hubei Province, situated in the “waist of the Yangtze River”, is the core region of the Yangtze River Economic Belt, boasting a well-developed shipping industry [2]. Based on the Statistical Bulletin of Hubei Province’s National Economic and Social Development, the cargo throughput of ports in the province has been increasing year by year (as shown in Figure 1). In 2023, the total cargo throughput of ports in the province reached 690 million tons, representing a year-on-year increase of 22.8%. The container throughput of ports was 3.298 million twenty-foot equivalent units (TEUs), showing a year-on-year increase of 5.5% [3]. To respond to China’s “dual-carbon” strategy and achieve the carbon emission reduction targets outlined in Hubei Province’s 14th Five-Year Plan, promoting ship decarbonization has become of particular significance. Among various measures, the adoption of clean power fuels is an effective solution.

At present, ammonia (NH_3_) and hydrogen (H_2_) are two clean fuels that have drawn significant attention. They are harmless to the environment after complete combustion [4,5,6]. However, the combustion of ammonia presents several challenges, such as slow combustion speed, high auto-ignition temperature, and narrow flammability limits [7,8,9]. Therefore, ammonia is usually used in combination with hydrogen to improve the combustion characteristics of the fuel. For example, Lhuillier et al. [10] found that adding 15% hydrogen to ammonia fuel can significantly shorten the start-up time. The research by Wang et al. [4] also indicated that incorporating hydrogen into ammonia can remarkably enhance the combustion performance of ammonia. These studies have demonstrated the feasibility of ammonia–hydrogen blended fuels in marine engines. There are mainly two ways to supply hydrogen on ships [11]. One is to pre-store hydrogen through hydrogen storage technologies. The other is to adopt on-line ammonia cracking for hydrogen production technology, which means decomposing hydrogen in real time during the operation of the ship. Although hydrogen storage technologies can provide a stable supply of hydrogen, their safety concerns and high transportation costs limit large-scale applications [12]. In contrast, the on-line ammonia cracking for hydrogen production technology can accurately adjust the hydrogen production according to the actual needs of the ship, avoiding the high risks and costs associated with traditional hydrogen storage [13]. This technology not only improves the conversion rate of ammonia but also provides a new solution for the green transformation of ships. At present, the ammonia cracking for hydrogen production technology is developing in a diversified manner, covering various technical routes such as thermal catalysis [14,15,16], electrocatalysis [17], photocatalysis [18], the non-thermal plasma method [19], and the non-thermal plasma catalysis method [20]. Among them, the non-thermal plasma catalysis method, as an emerging technology, has gradually attracted the attention of researchers [21,22,23]. It has the advantages of high efficiency, low cost, and mild operating conditions.

In view of this, the non-thermal plasma catalysis method can realize on-line hydrogen production by NH_3_ decomposition under mild conditions. However, for marine ammonia engines, there are still some challenges when using the waste heat of their exhaust gas to provide energy for the on-line hydrogen production technology of plasma-catalytic ammonia decomposition. Firstly, the exhaust gas temperature conditions of marine engines fluctuate due to various factors such as the navigation state and load changes, requiring the ammonia decomposition hydrogen production system to have a high degree of temperature adaptability and rapid response ability [24]. How to accurately regulate the reaction conditions (such as reaction temperature and pressure) of ammonia decomposition for hydrogen production to further improve the ammonia conversion rate has become a key bottleneck restricting the wide application of this technology [25,26]. In synergistic ammonia decomposition for hydrogen production by non-thermal plasma and catalyst, the improvement in the ammonia conversion rate directly affects the overall energy efficiency and emission performance of marine ammonia engines [27]. On the other hand, the ratio of different feed gases, especially the ratio of NH_3_ to other gases (such as N_2_), directly affects the ammonia conversion efficiency and hydrogen production of the non-thermal plasma catalytic reaction. The composition change in the feed gas may change the characteristics of plasma discharge, thus affecting the reaction mechanism. Therefore, the optimization of its reaction process parameters is a key issue that needs to be solved urgently. In addition, further research should also focus on the in-depth analysis of the reaction mechanism, including sensitivity analysis and reaction path analysis. Sensitivity analysis can reveal the sensitivity of each reaction parameter to the ammonia conversion rate, helping to optimize the reaction conditions and catalyst design. Reaction path analysis helps to clarify the detailed reaction path of ammonia decomposition under non-thermal plasma catalysis, providing a more comprehensive understanding from the initial excitation of the reaction to the final hydrogen generation process, thus guiding the regulation of the reaction system in practical applications.

In summary, this study employed a zero-dimensional plasma modeling approach to establish a numerical model for non-thermal plasma-catalyzed ammonia-to-hydrogen production. Additionally, the influencing factors (reaction temperature, pressure, and the ratio of N_2_/NH_3_ in the feed gas) and reaction mechanism pathways of the on-line ammonia-to-hydrogen production reaction in marine engines were analyzed.

## 2. Plasma Reaction Kinetics Modeling

The interactions between active particles such as energetic electrons, ions, and radicals in nanosecond-pulsed discharge (NPD) plasma and NH_3_ molecules involve multiple reaction steps and intermediate products. The evolution characteristics of these steps and products over time are difficult to directly observe and measure through experiments [28]. Most importantly, when a catalyst is introduced, the plasma and the catalyst are in the same region of the same reactor, and a synergetic effect will occur between them [28], which further increases the complexity of the reaction. Therefore, a zero-dimensional plasma chemical model was established using the ZDPlasKin solver [29]. (In the model, it is assumed that the gas temperature remains constant throughout the reaction process, with the energy released or absorbed during the reaction being neglected. Unless otherwise specified, the gas temperature is set at 300 K.) Starting from the complex reaction system, this model reveals the chemical process of the NPD plasma-catalyzed ammonia-to-hydrogen reaction and optimizes its process parameters.

### 2.1. Nanosecond-Pulsed Plasma Model

#### 2.1.1. Chemical Composition

**Figure 2:**
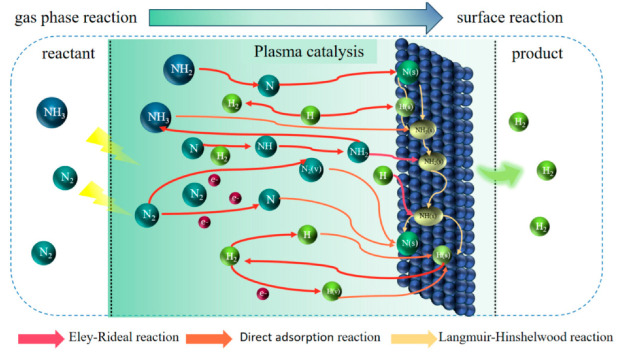
Schematic diagram of plasma-catalyzed ammonia decomposition.

The feed gas input to the model is N_2_/NH_3_, and the model includes two types of chemical reactions: gas-phase reactions and surface reactions (see Figure 2). Gas-phase reactions involve inelastic collisions between high-energy electrons and feed gas molecules (NH_3_, N_2_) in a nanosecond pulse discharge plasma environment, generating a series of highly reactive species. These reactive species, such as excited-state molecules, free radicals, and other intermediates, undergo further reactions in the gas phase, ultimately producing H_2_ [28]. Surface reactions involve the adsorption of feed gas molecules (NH_3_, N_2_) onto the catalyst surface, where they interact with active sites and subsequently react to form H_2_ [30]. Therefore, the zero-dimensional plasma dynamics model includes ground-state and excited-state N_2_ and H_2_ molecules and N and H atoms, as well as corresponding free radicals, ions, and surface-adsorbed species, as detailed in Table 1.

#### 2.1.2. Reaction Mechanism

If one wants to solve for the time-evolution characteristics of various particles during the ammonia decomposition for hydrogen production through the NPD plasma chemical model composed of the reaction mechanisms of plasma and catalyst, it is necessary to first calculate the reaction rate coefficients involved in its reaction mechanism [31]. Firstly, the reaction rate coefficients for the electron collision reactions are obtained by the Bolsig+ solver [32] according to the Boltzmann equation, combined with the electron collision cross-section data and electron energy; the specific contents are shown in Table 2. In addition to these, the model also takes into account reaction types such as charge exchange, Penning ionization, ion–ion recombination, electron–ion recombination, electron–ion dissociative recombination, wall-collision relaxation, three-body reactions, vibration-translation (V-T) relaxation, vibration-vibration (V-V) energy exchange, surface adsorption, and dissociation [28,31]. Among them, the rate coefficients for V-V and V-T relaxation reactions can be determined using the calculation formulae for k_VV_ and k_VT_ as proposed in reference [33] (for detailed formulae, please refer to reference [33]). The rate coefficients of wall-collision relaxation and surface reactions are obtained by calculating the wall-collision probability and the sticking probability. The specific calculation details can be found in published research [33,34,35]. However, due to the lack of sticking probabilities for each reaction mechanism of gas-phase particles on the catalyst surface [36], the metal Fe catalysts are described in this study without special emphasis. Generally speaking, the plasma-catalyzed reaction mechanism for ammonia-to-hydrogen production is a detailed and complex reaction set, covering up to 47 different species and 490 elementary reactions. These species and reactions together form the core framework of the plasma kinetics mechanism, providing a solid foundation for in-depth exploration and understanding of the plasma-assisted ammonia decomposition process for hydrogen production.

## 3. Results and Discussion

### 3.1. Model Verification

**Figure 3:**
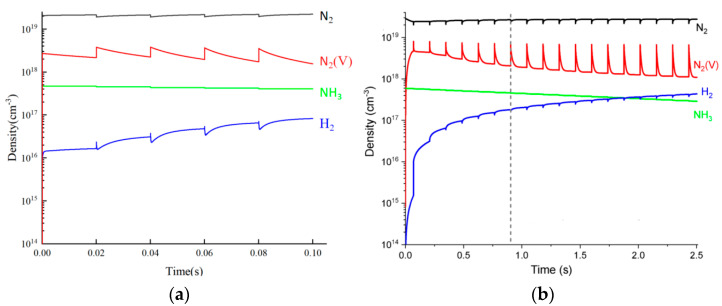
Comparison of the evolution characteristics of the species number density: (**a**) the model NPD plasma-catalyzed ammonia decomposition for hydrogen production; (**b**) the plasma-catalyzed ammonia decomposition model in Ref. [36] (Andersen et al., 2023) under CC-BY license.

Ammonia decomposition and ammonia synthesis are reversible reactions. Based on the plasma ammonia synthesis reaction mechanism of Veer et al. [34], and due to the lack of electron collision cross-section data for the reactions e + NH_3_ => e + NH_2_ + H and e + NH_3_ => e + NH + H in the existing database, they were replaced by the electron collision cross-section data of the reactions e + NH_3_ => e + NH_3_(e1) and e + NH_3_ => e + NH_3_(e2), respectively [31,38]. The purpose of this is to fill in the lack of NH_3_ electron reaction data in the mechanistic model, thus further refining the mechanistic model. The NPD plasma-catalyzed ammonia decomposition reaction for hydrogen production involves hundreds of reaction steps, and the calculation step size must be very small (≤1 × 10^−10^ s). Therefore, the calculation time was reduced, and the evolution characteristics of the particle number density within 0.1 s were verified, as shown in Figure 3. Figure 3a,b show the calculation results of the research model and the plasma-catalyzed ammonia decomposition model of Andersen et al. [36], respectively.

### 3.2. Analysis of Influencing Factors

#### 3.2.1. Reaction Temperature

**Figure 4:**
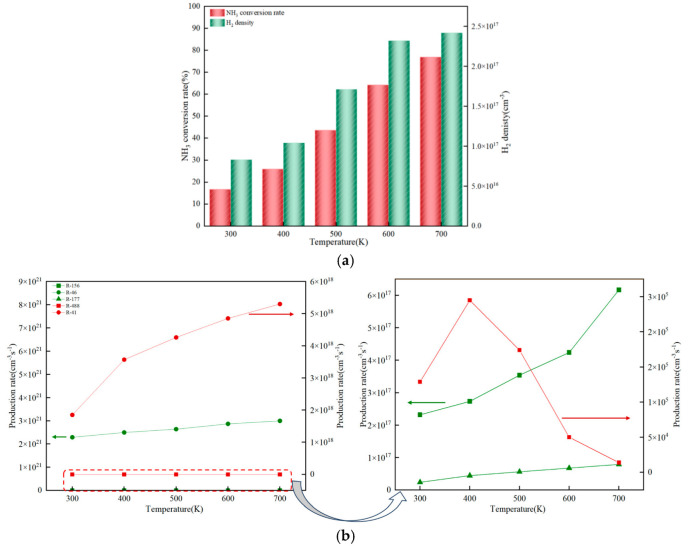
Influence of temperature on ammonia decomposition efficiency and yield. (**a**) Efficiency of hydrogen production from ammonia at different temperatures. (**b**) Yield of gas-phase and surface reactions for ammonia decomposition at the same temperature (1 bar).

The reaction process of ammonia synthesis is an exothermic process. As its reverse reaction, ammonia decomposition is an endothermic process. Therefore, as one of the important characterization parameters, the gas temperature plays a crucial regulatory role in NPD plasma-catalyzed ammonia decomposition (Figure 4). It not only affects the activity of the catalyst but also influences the plasma discharge characteristics, thereby changing the efficiency of ammonia-to-hydrogen production, as shown in Figure 4a. More specifically, the conversion rate of NH_3_ and the density of H_2_ show an increasing trend with the rise in the reaction temperature. This is because ammonia decomposition is an endothermic reaction, and an increase in temperature is beneficial for the shift in the equilibrium and promotes the production of H_2_ from NH_3_. Figure 4b shows the reaction yields of the gas-phase and surface reactions that mainly produce H_2_. The corresponding reaction equations are shown in Table 3. In the gas-phase reactions, the reaction yields of most reactions increase to varying degrees with the increase in temperature. In the surface reactions, the reaction yield of R-488 doubles in the range of 300–400 K, which means that at high temperatures, the activity of the catalyst is enhanced and H_2_ is more easily desorbed [39]. However, when the temperature rises from 400 K to 700 K, the reaction yield of R-488 shows a downward trend. This may be because the activity of particles in the ammonia-to-hydrogen reaction continuously increases with the rise in temperature, which leads to an increasing number of other particles competing with H(s) adsorbed on the active sites of the catalyst, thus resulting in a decrease in the reaction yield of R-488. Therefore, appropriately increasing the temperature is beneficial for the progress of the ammonia decomposition reaction.

#### 3.2.2. Reaction Pressure

**Figure 5:**
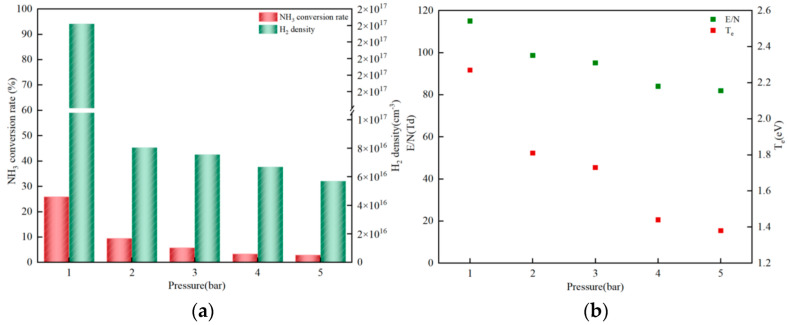
Effect of pressure on hydrogen production from ammonia efficiency, maximum E/N, and T_e_. (**a**) Efficiency of hydrogen production from ammonia at different pressures. (**b**) Maximum reduced electric field and electron temperature at different pressures (400 K).

The reaction pressure refers to the force exerted by the gas inside the reactor. As explored in [40], the influence of reaction pressure is significant during the ammonia decomposition process. Therefore, this paper conducts research on the efficiency of hydrogen production from ammonia decomposition under different reaction pressures (Figure 5). Figure 5a shows the efficiency of ammonia decomposition-to-hydrogen production under the coupling of NPD plasma and catalyst as a function of reaction pressure. With the increase in pressure, both the conversion rate of NH_3_ and the number density of H_2_ show a downward trend. Specifically, the conversion rate of NH_3_ drops from 25.88% to 2.92%, and the number density of H_2_ decreases by nearly three orders of magnitude. However, an increase in reaction pressure can effectively prolong the residence time of the gas in the reactor, enabling the gas to react fully and thus promoting the decomposition of NH_3_ into H_2_. In other words, an increase in pressure should theoretically improve the efficiency of ammonia decomposition-to-hydrogen production. So, what causes this decrease in efficiency? Subsequently, the reduced electric field E/N and electron temperature T_e_ during the ammonia decomposition process were investigated. It was found that both E/N and T_e_ decrease with the increase in reaction pressure (as shown in Figure 5b). The decrease in E/N and T_e_ inhibits the dissociation of gas molecules necessary for ammonia decomposition [31]. However, the increase in residence time is not sufficient to compensate for the impact of the decrease in the reduced electric field and electron temperature on ammonia decomposition for hydrogen production. In addition, lower pressure is not conducive to kinetic improvement [41]. Therefore, to maximize hydrogen production, the pressure needs to be optimized.

#### 3.2.3. Different Ratios of Feed Gases

**Figure 6:**
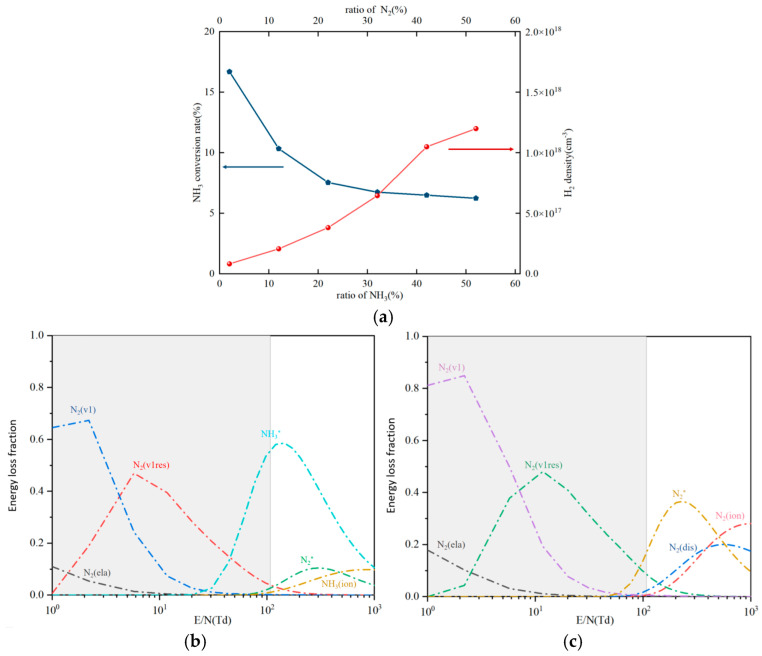
Effect of feed gas concentration on ammonia to hydrogen production efficiency and energy loss fraction. (**a**) Effect of NH_3_ concentration on NH_3_ conversion and H_2_ concentration (discharge frequency 3 kHz, pulse width 200 ns); (**b**) variation in energy loss fraction with E/N for the 48% N_2_/52% NH_3_ feed gas; (**c**) variation in the energy loss fraction with E/N for the 98% N_2_/2% NH_3_ feed gas (ela: elastic; dis: dissociation; ion: ionization; *: electronic excited state, 300 K).

Under normal temperature and pressure, the ratio of N_2_/NH_3_ was changed to investigate the effect of the feed gas concentration on plasma-catalyzed hydrogen production from NH_3_. Figure 6 shows the changes in H_2_ concentration and NH_3_ conversion rate with the increase in the proportion of NH_3_ in the feed gas. As can be seen from Figure 6a, the concentration of H_2_ shows an increasing trend. Although this increase is affected by the increase in the initial proportion of NH_3_, the relationship is non-linear [42]. However, during the increase in NH_3_ concentration, the NH_3_ conversion rate decreased from 16.7% to 6.25%. Since the discharge power in the ammonia decomposition system was not changed, with the increase in the number of reactive gas molecules, the unit energy density would decrease. A lower energy density would not cause more reactive gas molecules to decompose and convert [42]. According to the chemical equilibrium theory, ammonia decomposition and ammonia synthesis are reversible reactions. When the concentration of N_2_ in the reactor is increased, ammonia decomposition will be inhibited, thereby promoting the occurrence of the ammonia synthesis reaction. However, the results shown in Figure 6a are contrary to this. It can be seen that the plasma environment can, to a certain extent, hinder the occurrence of its reverse reaction. As shown in Figure 6b, in the range of 1–107 Td (1 Td = 10^−17^ V cm^2^) in this study, the vibrational excited state of N_2_ dominates during the plasma discharge process. With the increase in E/N, the electronic excited state of NH_3_ begins to become gradually important. For the feed gas with a ratio of 98% N_2_/2% NH_3_ (Figure 6c), when E/N is in the range less than 43 Td, the energy loss mechanism does not change significantly. However, in the range of 43-107 Td, the main energy loss pathway is the electronic excited state of N_2_. This transition means that increasing the proportion of N_2_ in the feed gas is beneficial for promoting the generation of the electronic excited state of N_2_, thereby promoting the decomposition of NH_3_ (such as N_2_(A^3^) + NH_3_ => N_2_ + NH_2_ + H) [32]. It is worth noting that increasing the proportion of N_2_ in the mixed gas will increase the energy loss fraction of the vibrational state and elastic collision state of N_2_ below 43 Td by an order of magnitude, reducing the energy utilization efficiency. Therefore, although the introduction of the diluent gas N_2_ accelerates the conversion of NH_3_, it also increases the energy consumption at the same time.

### 3.3. Reaction Mechanism Analysis

#### 3.3.1. Sensitivity Analysis

**Figure 7:**
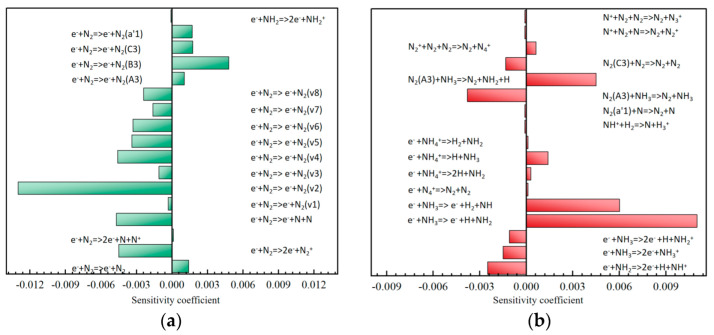
Sensitivity coefficients of NH_3_ at atmospheric pressure (300 K, 3 kHz frequency). (**a**) sensitivity coefficients for electron collision reaction; (**b**) sensitivity coefficients for electron collision reaction and heavy particle reaction.

Multiple intermediate reaction steps are involved in the plasma-catalytic process of ammonia-to-hydrogen production, and these reaction steps have different impacts on ammonia decomposition for hydrogen production. By calculating the sensitivity coefficients of the selected species, the degree of influence of each reaction step on the decomposition or generation of the species can be quantitatively evaluated, and then the key reaction steps can be identified. To explore the sensitivity of each reaction to NH_3_ decomposition, the species considered in this study was NH_3_, and the calculated sensitivity coefficients are presented in Figure 7. To reduce the time cost and improve the clarity, the vibrational energy exchange process was not considered, and only the reactions with S > ±1.0 × 10^−4^ were shown. A negative sensitivity coefficient indicates that the corresponding reaction inhibits the decomposition of the species, and vice versa. As can be observed from Figure 7b, within a pulse cycle, although the feed concentration of N_2_ is much higher than that of NH_3_, the main reaction promoting NH_3_ decomposition at room temperature is the dissociation reaction (R1) in which the N-H bond is broken due to the collision between electrons and NH_3_, rather than the dissociation of NH_3_ by the electronically excited state N_2_(A^3^) of N_2_ (R2). This indicates that the rate-limiting step of NH_3_ decomposition is the dissociation reaction of the collision between electrons and NH_3_(R1). Interestingly, for the plasma-catalytic process of NH_3_ for hydrogen production, the generation of the vibrational excited state of N_2_ shows negative sensitivity. Among them, the formation of N_2_(v2) has a significant inhibitory effect on NH_3_ decomposition. Since the reaction R3 accounts for 73% of the consumed N_2_, more N_2_ is consumed, and the electronically excited-state N_2_(A3) generated by the reaction R4 will be severely reduced, further inhibiting the decomposition of NH_3_. However, in Figure 7a, the generation of the electronically excited state of N_2_ shows positive sensitivity. This is because N_2_ (A3) promotes the conversion of NH_3_ through the dissociation reaction (R5), and its contribution rate is 53%. Therefore, an increase in the concentration of N_2_(a’1) in the reactor will increase the concentrations of N_2_(B3) and N_2_(C3) (R6, R7), and an increase in the concentrations of N_2_(B3) and N_2_(C3) (R8-14) will in turn increase the concentration of N_2_(A3), thereby accelerating the decomposition of NH_3_. This demonstrates the importance of the electronically excited state of N_2_ for NH_3_ decomposition to produce hydrogen. In addition, the ionization reactions of the electron collisions with and NH_3_ and N_2_, called R15 and R16, are the main sources of electrons. The more electrons generated, the faster the decomposition rate of NH_3_. However, the sensitivity coefficients shown by the two reactions in Figure 7 are negative. Since the electron collision reactions obtain the concentration C’ by doubling their collision cross-sections, the selection of the electron reaction collision cross-section is very important for NH_3_ decomposition.

e^−^ + NH_3_ => e^−^ + NH_2_ + H (R1)

e^−^ + NH_3_ => e^−^ + NH + H_2_ (R2)

N_2_(v2) + N_2_ => N_2_(v1) + N_2_(v1) (R3)

e^−^ + N_2_ => e^−^ + N_2_(A3) (R4)

N_2_(A3) + NH_3_ => N_2_ + NH_2_ + H (R5)

N_2_(a’1) + N_2_ => N_2_(B3) + N_2_ (R6)

N_2_(a’1) + N_2_ => N_2_(C3) + N_2_ (R7)

N_2_(B3) + H_2_ => N_2_(A3) + H_2_ (R8)

N_2_(B3) + N_2_ => N_2_(A3) + N_2_ (R9)

N_2_(B3) + N => N_2_(A3) + N (R10)

N_2_(B3) + N_2_ => N_2_(A3) + N_2_(v6) (R11)

N_2_(B3) + N_2_(v1) => N_2_(A3) + N_2_(v7) (R12)

N_2_(B3) + N_2_(v2) => N_2_(A3) + N_2_(v8) (R13)

N_2_(C3) + N_2_ => N_2_(B3) + N_2_(A3) (R14)

e^−^ + NH_3_ => 2e^−^+ NH_3_^+^ (R15)

e^−^ + N_2_ => 2e^−^+ N_2_^+^ (R16)

NH_2_ + H => NH + H_2_ (R17)

N + H_2_(v3) => NH + H (R18)

NH + H => H_2_ + N (R19)

e + N_2_ => e +N + N (R20)

N + NH_2_ => N_2_ + 2H (R21)

H + NH_2_ => H_2_ + NH (R22)

H + H(s) => H_2_ (R23)

H(s)+ H(s) => H_2_ (R24)

H_2_ + N_2_(a’1) => 2H + N_2_ (R25)

H_2_+ N_2_(v) => N_2_(v−1)+ H_2_(v) (R26)

H + N(s) => NH(s)(R27)

H(s) + NH(s) => NH_2_(s) (R28)

NH_2_(s)+ H(s) => NH_3_ (R29)

NH => NH(s)(R30)

N + H(s) => NH(s)(R31)

H + N(s) => NH(s)(R32)

NH(s)+ H(s) => NH_2_(s) (R33)

NH_2_ => NH_2_(s) (R34)

#### 3.3.2. Reaction Path Analysis

**Figure 8:**
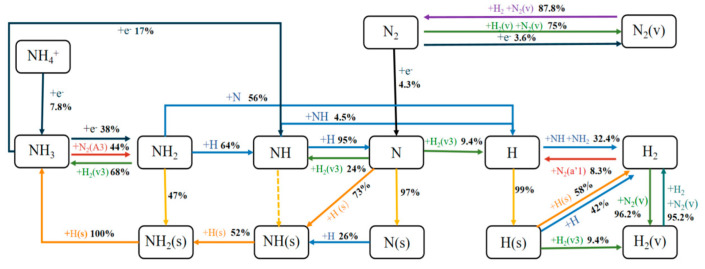
Reaction pathways of hydrogen production from ammonia catalyzed by plasma under atmospheric pressure, 300 K and frequency of 3 kHz (blue: reaction with free radicals, green: reaction with vibratory excited states, red: reaction with electron excited states, navy blue: electron collision reaction, yellow: direct adsorption reaction, orange: surface reaction, purple: the total reaction of the vibrational excited state and free radicals; dotted line indicates the resulting contribution <0.1%).

Based on the Pumpkin 1.4 software [43], this study simplified the chemical reaction pathways in the NPD plasma-catalytic ammonia-to-hydrogen production model and calculated the generation and consumption ratios of various species. The main reaction pathway diagram is shown in Figure 8. This reaction pathway was analyzed within the total gas residence time, providing insights into the interactions among various particles such as free radicals, molecules, and ions during the ammonia decomposition process for hydrogen production. As one of the feed gas molecules, NH_3_ is dissociated by electrons to generate NH_2_ and NH. Most of the NH_3_ is consumed through R5 to produce the species NH_2_. Besides R2, the main formation pathways of NH also include R17 and the reaction between molecules and atoms caused by N + H_2_(v3) => NH + H (R18). Their contribution rates to NH are 64% and 24%, respectively. The reaction R19, which occurs when the species NH collides with H atoms, contributes 95% of N and is the main source of N atoms. In addition, only a small part of N is formed by the collision of another feed gas molecule N_2_ with electrons (R20). However, most of the loss of N_2_ occurs through the so-called vibrational-translational (V-T) reaction, i.e., N_2_ + H_2_(v)/N_2_(v) => N_2_(v) + H_2_(v − 1)/N_2_(v − 1) (the reactions are shown in the Supplementary Materials) to form the vibrationally excited-state N_2_(v). Eventually, nearly 90% of N_2_(v) decays to N_2_. Therefore, under the reaction conditions and residence time considered in this study, the vibrationally excited pathway does not promote the dissociation of N_2_ [44]. The generated NH_2_, NH, and N species all contribute to the production of H atoms, among which R21 has the largest contribution rate, with a value of 56%. Overall, there are two main consumption pathways for H atoms, both of which are involved in the process of producing H_2_. One pathway is the reaction of H with free radicals NH and NH_2_ to form H_2_ (R19, R22). The other is that H is adsorbed on the catalyst to become adsorbed H(s) and H(s) undergoes a recombination reaction with H(s) and H to produce H_2_ (R23, R24). Their contribution rates are 58% and 42%, respectively. However, the H_2_ formed later is consumed by collisions with N_2_(a’1) and N_2_(v) undergoing the reactions R25 and R26. The formation of H_2_(v) quickly returns to the ground-state H_2_, although the vibrationally excited-state N_2_(v) consumes a large proportion of H_2_ (the reactions are shown in the Supplementary Materials) up to 96.2%. This confirms that the vibrationally excited channel does not promote the dissociation of H_2_ either.

In the plasma-catalytic process of ammonia decomposition, not only gas-phase reactions but also a series of surface reactions occur, initiated by surface species. It is noteworthy that compared with the species H(s), the reactions (R27-29) initiated by surface species such as N(s), NH(s), and NH_2_(s) have a negative contribution to the decomposition of NH_3_ to produce H_2_ and can lead to the synthesis of NH_3_ [36]. Specifically, as can be seen from Figure 8, most of the adsorbed states of various species on the catalyst surface originate from the direct adsorption of gas-phase species, except for NH(s) and NH_2_(s). For NH(s), the direct adsorption of NH(R30) contributes very little to its formation, less than 0.1%. Instead, NH(s) mainly comes from R31 and R32. The formed NH(s) will react step by step with H(s) to generate NH_2_(s) (R33) within the residence time and finally produce NH_3_ (R29). In this process, for NH_2_(s), the hydrogenation reaction of NH(s) (R28) contributes 52% to its formation, which is slightly greater than its own direct adsorption (47%) (R34). Overall, the decomposition of NH_3_ mainly occurs in gas-phase reactions. Moreover, compared with feeding pure NH_3_, when N_2_ is incorporated into the feed gas, N_2_ collides with electrons to produce the reaction e^−^+N_2_ => e^−^ + N_2_(A3), generating N_2_(A3). The R3 initiated by N_2_(A3) is the main reaction for decomposing NH_3_, and its contribution rate is greater than that of the two dissociation reactions generated by electron collisions (R1, R2). At the same time, the N atoms produced by the collision of N_2_ with electrons (R20) not only affect the conversion of NH_2_ but also contribute to the formation of H [36]. Therefore, the addition of an appropriate amount of N_2_ is beneficial for the decomposition of NH_3_ to obtain H_2_.

## 4. Calculation Method

### 4.1. Calculation of the Particle Number Density and Reduced Electric Field Strength

#### 4.1.1. Calculation of Particle Number Density

The plasma-catalyzed reaction process of NH_3_ decomposition is numerically simulated using the solver ZDPlaKin [12]. This solver explores the average behavior of the particle time-evolution by calculating the continuity equation of the number density n of each particle *i*. The equation is as follows [38]:

(1) $$\frac{dn_{i}}{dt}=\sum_{j}a_{j,i}k_{j}\prod_{s}n_{s}$$

In the equation, $a_{j,i}$ is the stoichiometric coefficient of a given particle *i* in reaction *j*, $k_{j}$ is the reaction rate coefficient ($k_{j}$ of heavy particle reaction is generally a function related to $T_{gas}$), and *s* is the species that collides with particle *i*.

#### 4.1.2. Calculation of the Reduced Electric Field Strength

During the nanosecond-pulsed plasma discharge process, charged particles are accelerated by the electric field intensity E and undergo directed motion to form current density *J*. As these charged particles move, they collide with neutral particles, transferring the kinetic energy acquired from the electric field to the neutral particles. This increases the thermal motion of the neutral particles, thereby generating heat, specifically Joule heating *J*∙*E* (*J* = σ∙*E* [39], where σ is conductivity). Consequently, the formula for the rate at which electrical energy consumed per unit effective volume in the reactor is converted into heat energy can be expressed as follows [31]:

(2) $$\frac{\mathrm{dP}}{dV}=JE=\sigma E^{2}$$

In Equation (2), P is the input discharge power and the unit is W.

Given that the discharge power of the input nanosecond-pulsed plasma is a time-dependent function p(*t*), the corresponding power density can be expressed as p(*t*) = p(*t*)/V. Therefore, the expression [30,31,36] for the reduced electric field E/N entered in the ZDPlasKin solver is as follows (where *E*/*N* varies with time in the form of a triangular pulse):

(3) $$\frac{E}{N}=\frac{1}{N}\sqrt{\frac{p(t)}{\sigma }}$$

In Formula (3), *N* represents the number density of all neutral particles, with units of cm^−3^.

### 4.2. Calculation of Ammonia Conversion Rate and Sensitivity Coefficient

The activity of the NPD plasma-catalyzed ammonia decomposition reaction for hydrogen production is mainly evaluated using the ammonia conversion rate. The detailed calculation formula is as follows:

(4) $$X_{NH_{3}}=\left(\frac{n_{NH_{3}}^{0}-n_{NH_{3}}}{n_{NH_{3}}^{0}}\right)\times 100\%$$

In the equation, $n_{{NH}_{3}}^{0}$ is the initial concentration and $n_{{NH}_{3}}$ is the concentration at the reactor outlet.

A study on the sensitivity of ammonia (NH_3_) decomposition for hydrogen production was carried out to gain a deeper understanding of the role of each reaction in the NPD plasma-catalyzed ammonia-to-hydrogen process. The sensitivity coefficient formula is defined as follows:

(5) $$S=\frac{\mathrm{lg}(C/C^{\prime })}{\mathrm{lg}(k_{j}^{\prime }/k_{j})}$$

In the equation, *S* is the sensitivity coefficient, *C* is the initial concentration of the selected particles, $k_{j}$ is the reaction rate coefficient of the *j*th reaction, and *C′* is the concentration of the species after doubling the reaction rate coefficient ($k_{j}^{\prime }$) of the *j*th reaction. *C′* is calculated by doubling the collision cross-section for electron collision reactions [31].

## 5. Conclusions

Among the input parameters of this numerical model, both the NH_3_ conversion rate and the H_2_ number density show an increasing trend with the increase in the reaction temperature. At 700 K, the NH_3_ conversion rate and the H_2_ number density are close to 80% and 2.5 × 10^17^ cm^−3^, respectively. The discharge pressure affects the reduced electric field E/N and the electron temperature T_e_ during the ammonia decomposition process for hydrogen production. An increase in the discharge pressure can reduce E/N and T_e_, thereby inhibiting the dissociation of gas molecules necessary for ammonia decomposition and slowing down the decomposition of NH_3_ into H_2_. In addition, changes in the N_2_/NH_3_ ratio of the feed gas can alter various sensitivity coefficients and reaction pathways of the ammonia-to-hydrogen reaction. The higher the N_2_/NH_3_ ratio, the greater the ammonia conversion rate. This is because increasing the proportion of N_2_ in the feed gas is conducive to promoting the generation of the electronically excited state of N_2_, which in turn promotes the decomposition of NH_3_ (such as N_2_(A3) + NH_3_ => N_2_ + NH_2_ + H). At the same time, an increase in the proportion of N_2_ in the mixed gas can increase the energy loss fraction of the vibrational state and elastic collision state of N_2_ below 43 Td by an order of magnitude, reducing the energy utilization efficiency. Therefore, although the introduction of the diluent gas N_2_ accelerates the conversion of NH_3_, it also increases the energy consumption.

## Figures and Tables

**Table 1:** Particle species in the model.

Particle Species	Chemical Formula
Ground-state molecule	N_2_, H_2_, NH_3_
Atom/radical	N, H, NH, NH_2_, NH_3_, N_2_H, N_2_H_2_, N_2_H_3_, N_2_H_4_
Excited-state molecule	N_2_ (v1), N_2_ (v2), N_2_ (v3), N_2_ (v4), N_2_ (v5), N_2_ (v6), N_2_ (v7), N_2_ (v8), H_2_ (v1), H_2_ (v2), H_2_ (v3), N_2_ (a’^1^), N_2_ (A3), N_2_ (B3), N_2_ (C3), N_2_ (^2^D), N_2_ (^2^P)
Ion	$N^{+}$, $N_{2}^{+}$, $N_{3}^{+}$, ${NH}^{+}$, ${NH}_{2}^{+}$, ${NH}_{3}^{+}$, ${NH}_{4}^{+}$, $H^{+}$, $H_{2}^{+}$, $H_{3}^{+}$, ${N_{2}H}^{+}$, e^−^, H^−^
Surface-adsorbed particle	N (s), H (s), NH (s), NH_2_ (s), NH_3_ (s)

Note: X(s) is the surface adsorption site.

**Table 2:** Collision cross-section data and reaction rate coefficient sources of particle reaction type.

Particle Reaction Types	Source of Collision Cross-Section Data and Reaction Rate Coefficient ^a^
The dissociation reaction of NH_3_ colliding with electrons	Ref. [37]
The elastic collision reaction of NH_3_ with electrons	Morgan database
The ionization reaction of NH_3_ colliding with electrons	Morgan database
The vibrational reaction of N_2_ colliding with electrons	Phelps database
The electron excitation reaction of N_2_ colliding with electrons	Phelps database
The elastic collision reaction of N_2_ colliding with electrons	Phelps database
The ionization reaction of N_2_ colliding with electrons	Itikawa database
The dissociation reaction of N_2_ collision with electrons	Ref. [37]
The elastic collision reaction of H_2_ colliding with electrons	Itikawa database
The vibrational reaction of H_2_ colliding with electrons	Phelps database
The electron excitation reaction of H_2_ colliding with electrons	Itikawa database
The dissociation reaction of H_2_ colliding with electrons	Itikawa database
The ionization reaction of H_2_ colliding with electrons	Itikawa database
The electron excitation reaction of N colliding with electrons	Ref. [37]
The elastic collision reaction of N colliding with electrons	Ref. [37]
The ionization reaction of N colliding with electrons	Ref. [37]
The elastic collision reaction of H colliding with electrons	Ref. [37]
The ionization reaction of H colliding with electrons	Ref. [37]

Note: ^a^: the reaction rate coefficient in cm^3^/s.

**Table 3:** Main reactions of pulsed plasma-catalyzed hydrogen production from ammonia.

Reaction Number	Reaction Equation
R-156	N + NH_2_ => H_2_ + N_2_
R-46	e + NH_3_ => e + NH + H_2_
R-177	NH + NH => H_2_ + N_2_
R-41	e + NH_2_ => e + N+H_2_
R-488	H(s) + H(s) => H_2_

## Data Availability

Data are contained within the article.

## Supplementary Materials

The following supporting information can be downloaded at: https://www.mdpi.com/article/10.3390/molecules30051054/s1. The chemical kinetic file of the reaction of plasma-catalyzed ammonia decomposition for hydrogen production.
